# Trastuzumab beyond progression in patients with HER2-positive advanced gastric adenocarcinoma: a multicenter AGEO study

**DOI:** 10.18632/oncotarget.20711

**Published:** 2017-09-08

**Authors:** Juliette Palle, David Tougeron, Astrid Pozet, Emilie Soularue, Pascal Artru, Florence Leroy, Olivier Dubreuil, Matthieu Sarabi, Nicolas Williet, Sylvain Manfredi, Jerome Martin-Babau, Christine Rebischung, Meher Ben Abdelghani, Ludovic Evesque, Johann Dreanic, Vincent Hautefeuille, Samy Louafi, David Sefrioui, Francesco Savinelli, May Mabro, Benoit Rousseau, Cédric Lecaille, Olivier Bouché, Christophe Louvet, Thierry Lecomte, Franck Bonnetain, Julien Taieb, Aziz Zaanan

**Affiliations:** ^1^ Department of Gastroenterology and Digestive Oncology, European Georges Pompidou Hospital, Assistance Publique-Hôpitaux de Paris, Paris, France; ^2^ Department of Gastroenterology, Poitiers University Hospital, Poitiers, France; ^3^ Methodology and Quality of Life in Oncology Unit, INSERM UMR 1098, Besançon University Hospital, Besançon, France; ^4^ Department of Medical Oncology, Saint Antoine Hospital, Assistance Publique-Hôpitaux de Paris, Paris, France; ^5^ Department of Gastroenterology and Digestive Oncology, Jean Mermoz Hospital, Lyon, France; ^6^ Department of Cancer Medicine, Gustave Roussy Institute, Villejuif, France; ^7^ Department of Gastroenterology and Digestive Oncology, Pitié-Salpêtrière Hospital, Assistance Publique-Hôpitaux de Paris, Paris, France; ^8^ Department of Medical Oncology, Léon Bérard Center, Lyon, France; ^9^ Department of Gastroenterology and Digestive Oncology, Saint-Etienne University Hospital, Saint-Priest en Jarez, France; ^10^ Department of Gastroenterology and Digestive Oncology, Rennes University Hospital, Rennes, France; ^11^ Department of Oncology, Morvan University Hospital, Brest, France; ^12^ Department of Oncology, Groupe Hospitalier Mutualiste of Grenoble, Grenoble, France; ^13^ Department of Oncology, Paul Strauss Center, Strasbourg, France; ^14^ Department of Oncology, Antoine Lacassagne Center, Nice, France; ^15^ Department of Gastroenterology and Digestive Oncology, Cochin Hospital, Assistance Publique-Hôpitaux de Paris, Paris, France; ^16^ Department of Gastroenterology and Digestive Oncology, Amiens University Hospital, Amiens, France; ^17^ Department of Oncology, Oncology Federation of Essonne, Essonne, France; ^18^ Department of Digestive Oncology, Rouen University Hospital, Rouen, France; ^19^ Department of Medical Oncology, Saint-Joseph Hospital, Paris, France; ^20^ Department of Oncology, Foch Hospital, Suresnes, France; ^21^ Department of Oncology, Henri Mondor Hospital, Assistance Publique-Hôpitaux de Paris, Créteil, France; ^22^ Department of Gastroenterology and Digestive Oncology, Polyclinique de Bordeaux Nord, Bordeaux, France; ^23^ Department of Gastroenterology and Digestive Oncology, Reims University Hospital, Reims, France; ^24^ Department of Medical Oncology, Mutualiste Montsouris Institute, Paris, France; ^25^ Department of Gastroenterology and Digestive Oncology, Trousseau University Hospital, Tours, France; ^26^ Paris Descartes University, Sorbonne Paris Cité, Paris, France

**Keywords:** advanced gastric cancer, trastuzumab, HER2, second-line chemotherapy, beyond progression

## Abstract

**Introduction:**

Trastuzumab in combination with platinum-based chemotherapy is the standard first-line regimen in HER2-positive advanced gastric cancer. However, there are very few data concerning efficacy of continuing trastuzumab beyond first-line progression.

**Methods:**

This retrospective multicenter study included all consecutive patients with HER2-positive advanced gastric or gastro-esophageal junction (GEJ) adenocarcinoma who received a second-line of chemotherapy with or without trastuzumab after progression on platinum-based chemotherapy plus trastuzumab. Progression-free survival (PFS) and overall survival (OS) were estimated from the start of second-line chemotherapy using the Kaplan-Meier method and compared using log-rank test. The prognostic variables with *P* values ≤ 0.05 in univariate analysis were eligible for the Cox multivariable regression model.

**Results:**

From May 2010 to December 2015, 104 patients were included (median age, 60.8 years; male, 78.8%; ECOG performance status [PS] 0-1, 71.2%). The continuation (n=39) versus discontinuation (n=65) of trastuzumab beyond progression was significantly associated with an improvement of median PFS (4.4 versus 2.3 months; *P*=0.002) and OS (12.6 versus 6.1 months; *P*=0.001. In the multivariate analysis including the ECOG PS, number of metastatic sites and measurable disease, the continuation of trastuzumab beyond progression remained significantly associated with longer PFS (HR, 0.56; 95% CI, 0.35-0.89; *P*=0.01) and OS (HR, 0.47; 95% CI, 0.28-0.79; *P*=0.004).

**Conclusion:**

This study suggests that continuation of trastuzumab beyond progression has clinical benefit in patients with HER2-positive advanced gastric cancer. These results deserve a prospective randomized validation.

## INTRODUCTION

Gastric cancer is the fourth most commonly diagnosed cancer and the second most common cause of cancer-related deaths worldwide [1]. For patients with inoperable locally advanced or metastatic disease, systemic chemotherapy improves survival and quality of life compared with best supportive care alone [2]. In first-line treatment, doublet combination of platinum salts with fluoropyrimidine is considered as a standard of care [3, 4], and there remains controversy regarding the utility of adding anthracycline [5] or taxanes [6]. More recently, irinotecan-based chemotherapy has been suggested as a validated alternative therapy to platinum-based regimen [7, 8].

In HER2-positive advanced gastric or gastro-esophageal junction (GEJ) adenocarcinoma, which accounts for approximately 10% to 30% of tumors [9], the randomized phase III ToGA trial demonstrated a significant improvement in progression-free survival (PFS) and overall survival (OS) with the addition of trastuzumab to cisplatin and fluoropyrimidine regimens (median OS: 13.8 versus 11.1 months; hazard ratio [HR], 0.74; 95% confidence interval [CI], 0.60–0.91; *P*=0.005) [10]. Patients were eligible in this trial if their tumor samples were HER2-positive scored as 3+ on immunohistochemistry (IHC) or if they were positive in fluorescence in situ hybridization (FISH) [10]. In pre-planned exploratory analysis, the survival benefit of trastuzumab was stronger in the HER2-positive subgroup with IHC 3+ or 2+/FISH positive tumors (median OS: 16.0 versus 11.8 months; HR, 0.65; 95% CI, 0.51–0.83) [10]. More recently, two phase II studies have suggested that oxaliplatin was an interesting alternative drug instead of cisplatin in combination with fluoropyrimidine and trastuzumab [11, 12].

Nowadays, the strategy of second- and further-line treatment does not take into account the HER2 tumor status. In patients with an adequate condition status, second-line treatment may offer an improvement in OS and quality of life compared with best supportive care alone [7, 8]. The validated therapeutic options include taxanes (docetaxel or paclitaxel) [13] and irinotecan alone [14] or combined with 5-FU (FOLFIRI regimen) [8], if not used before. A randomized phase III trial directly comparing paclitaxel versus irinotecan has demonstrated similar efficacy for both regimens [15]. More recently, two randomized phase III trials have demonstrated that ramucirumab was associated with a survival benefit as a single agent compared to best supportive care alone [16], whereas ramucirumab in addition to paclitaxel was associated with a survival benefit compared with paclitaxel alone [17].

From the first- to second-line treatment of cancer, the cytotoxic drugs are generally discontinued upon disease progression leading to change to another chemotherapy regimen. However, resistance to cytotoxic drugs is not always applicable to biologic agents, such as bevacizumab in metastatic colorectal cancer [18]. In HER2-positive advanced breast cancer, studies have shown that after failure of trastuzumab-containing therapy, the continuing of trastuzumab beyond progression in combination with another regimen of chemotherapy was associated with improvement of clinical outcomes [19, 20]. This interesting therapeutic strategy of continuing trastuzumab beyond progression has been little evaluated in gastric cancer. Thus, in this current study, we evaluate the efficacy of continuing trastuzumab after failure of first-line platinum-based chemotherapy plus trastuzumab in patients with HER2-positive advanced gastric or GEJ adenocarcinoma.

## RESULTS

### Patient characteristics

**Figure 1:**
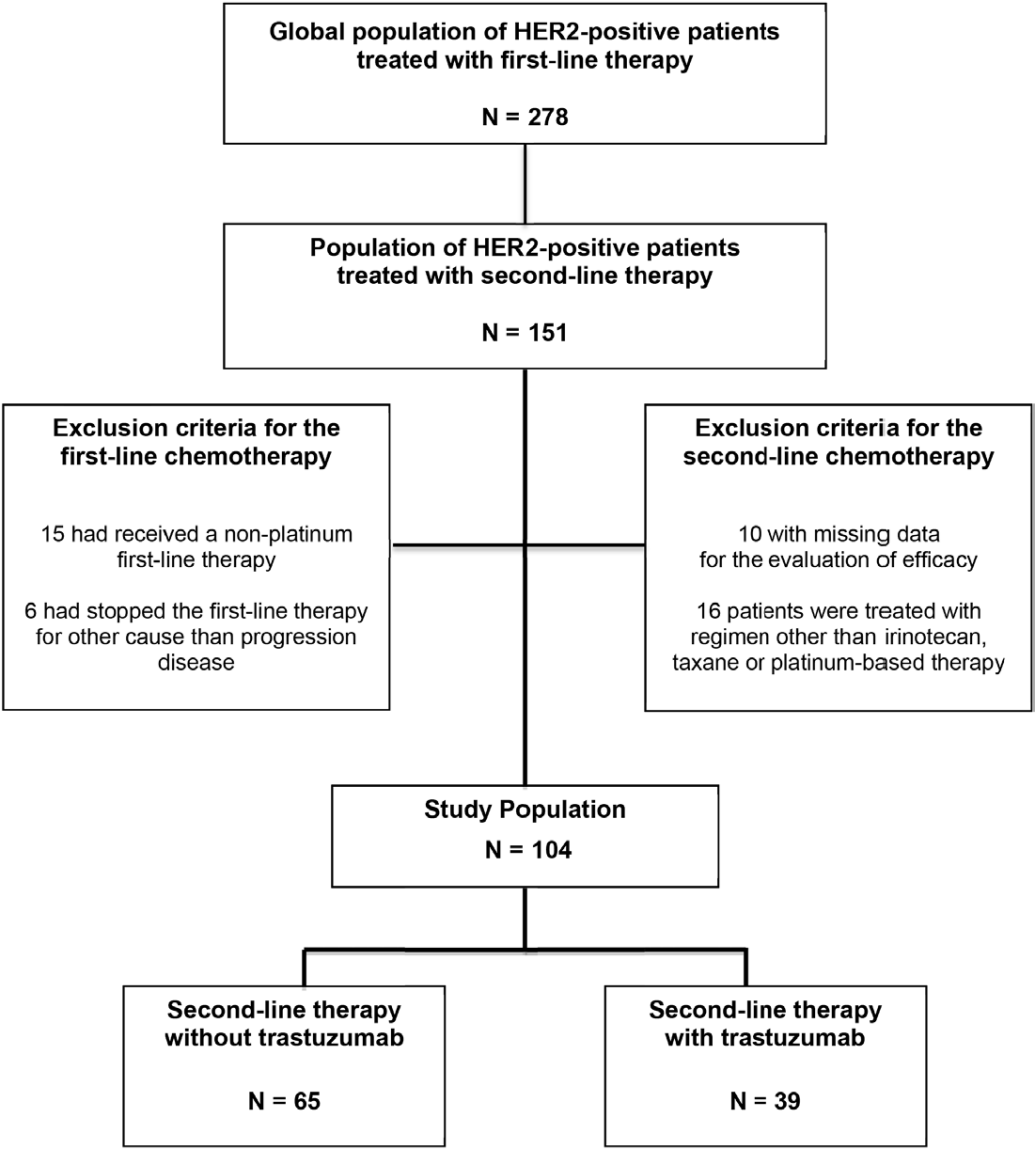
Flow Chart of study

Among 278 patients with HER2-positive advanced gastric or GEJ adenocarcinoma treated in first-line treatment, we screened 151 patients who have received a second-line treatment between May 2010 and December 2015 (Figure 1). Among these 151 patients, we excluded patients who had received a second-line regimen other than those specified for this study (n=16), and those for whom the evaluation of efficacy was not available (n=10). We also excluded patients who had received a non-platinum first-line therapy (n=15) or had stopped the first-line therapy for other cause than progression disease (n=6) (Figure 1). The study population therefore consisted of 104 patients (median age, 60.8 years; male, 78.8%; ECOG PS 0-1, 71.2%) with advanced (metastatic stage, 99%) gastric (45.2%) or GEJ (54.8%) cancer (Table 1). The median follow-up was 25.9 months (95% CI, 16.4–30.8).

**Table 1:** Clinical and pathological characteristics at baseline of the second-line of chemotherapy

	Overall Population	Second-line therapy without trastuzumab	Second-line therapy with trastuzumab	*P-value*
	N= 104	N = 65	N = 39	
**Age**				0.41
Mean	60.0	61.3	57.9	
Median [range]	60.8 [17.3–80.3]	59.0 [34.0-80.3]	61.1 [17.3-80.1]	
**Gender**				0.53
Male	82 (78.8%)	50 (76.9%)	32 (82.1%)	
Female	22 (21.2%)	15 (23.1%)	7 (17.9%)	
**ECOG PS**				0.81
0-1	74 (71.2%)	45 (69.2%)	29 (74.4%)	
≥ 2	23 (22.1%)	15 (23.1%)	8 (20.5%)	
Unknown	7 (6.7%)	5 (7.7%)	2 (5.1%)	
**Primary tumor site**				<0.0001
Gastro-esophageal junction	57 (54.8%)	25 (38.5%)	32 (82.1%)	
Stomach	47 (45.2%)	40 (61.5%)	7 (17.9%)	
**Histological type**				0.06
Intestinal	54 (51.9%)	34 (52.3%)	20 (51.3%)	
Diffuse or mixed	12 (11.6%)	4 (6.2%)	8 (20.5%)	
Other or unknown	38 (36.5%)	27 (41.5%)	11 (28.2%)	
**Tumor grade**				0.41
Well/Moderately differentiated	63 (60.6%)	37 (56.9%)	26 (66.7%)	
Poorly differentiated	29 (27.9%)	21 (32.3%)	8 (20.5%)	
Unknown or missing	12 (11.5%)	7 (10.8%)	5 (12.8%)	
**Extent of disease**				0.15
Locally advanced	1 (1.0%)	0 (0%)	1 (2.5%)	
Metastatic	103 (99.0%)	65 (100%)	38 (97.5%)	
**Measurable disease**	91 (87.5%)	55 (84.6%)	36 (92.3%)	0.32
**Number of metastatic sites**				0.03
0-1	32 (30.8%)	15 (23.1%)	17 (43.6%)	
≥2	72 (69.2%)	50 (76.9%)	22 (56.4%)	
**HER2 status**				0.16
IHC 3+	83 (79.8%)	49 (75.4%)	34 (87.2%)	
IHC 2+/FISH-positive	17 (16.4%)	14 (21.5%)	3 (7.7%)	
Unknown	4 (3.8%)	2 (3.1%)	2 (5.1%)	
**First-line chemotherapy regimen**				<0.0001
FP + oxaliplatin + trastuzumab	50 (48.1%)	21 (32.3%)	29 (74.4%)	
FP + cisplatin + trastuzumab	54 (51.9%)	44 (67.7%)	10 (25.6%)	
**Second-line chemotherapy regimen**				0.03
FOLFIRI	67 (64.4%)	48 (73.9%)	19 (48.7%)	
Taxane	23 (22.1%)	11 (16.9%)	12 (30.8%)	
FP + platinum salts	14 (13.5%)	6 (9.2%)	8 (20.5%)	

Abbreviations: IHC, immunohistochemistry; FISH, fluorescence in-situ hybridization; FP, Fluoropyrimidine

The trastuzumab was discontinued in second-line treatment for 65 patients (62.5%) and continued for 39 patients (37.5%). The most frequent second-line regimen was FOLFIRI (64.4%), whatever the association or not with trastuzumab. All patients received first-line chemotherapy based on trastuzumab in combination with fluoropyrimidine and cisplatin (51.9%) or oxaliplatin (48.1%).

The clinical and pathological characteristics did not differ significantly between the two groups of discontinuation versus continuing trastuzumab in second-line treatment, except for the primary tumor site (GEJ, 38.5% versus 82.1%, respectively; *P*<0.0001) and the number of metastatic sites (≥2 metastatic sites, 76.9% versus 56.4%, respectively; *P*=0.03) (Table 1). In addition, for patients who continued trastuzumab beyond progression, a first-line of oxaliplatin-based chemotherapy was more frequently administered (74.4%) than cisplatin-based chemotherapy (25.6%) (*P*<0.0001) (Table 1).

The other patient characteristics are summarized in Table 1. We observed a trend toward longer median PFS (HR, 0.76; 95% CI, 0.51-1.13; *P*=0.18) and a significant higher objective response rate (ORR) (57% versus 24%; *P*=0.001) in first-line treatment for patients who have continued as compared with those who have discontinued trastuzumab beyond progression.

### Tumor response

Tumor response was evaluated in 91 patients (87.5%) who had measurable disease. In the overall population, the ORR and disease control rate (DCR) in second-line treatment were 9.9% and 36.3%, respectively (Table 2). In comparison with patients who stopped trastuzumab in second-line chemotherapy, those who continued it had a trend toward higher ORR (16.7% versus 5.4%; *P*=0.08) and a significantly higher DCR (50.0% versus 27.3%; *P*=0.03) (Table 2).

**Table 2:** Analysis of tumor response and survival according to the continuation or not of trastuzumab from the second-line of chemotherapy

	Overall Population N = 104	Second-line therapy without trastuzumab N = 65	Second-line therapy with trastuzumab N = 39	*P-value*
**Tumor response**				
Complete response	3 (2.9%)	1 (1.5%)	2 (5.1%)	
Partial response	6 (5.7%)	2 (3.1%)	4 (10.3%)	
Stable disease	24 (23.1%)	12 (18.5%)	12 (30.8%)	
Progression disease	58 (55.8%)	40 (61.5%)	18 (46.1%)	
Not evaluable	13 (12.5%)	10 (15.4%)	3 (7.7%)	
Objective response rate ^*^	9.9%	5.4%	16.7%	0.08
Disease control rate ^*^	36.3%	27.3%	50.0%	0.03
**Median PFS, mo. (95% CI)**	2.7 (2.3-4.0)	2.3 (2.0-3.0)	4.4 (2.4-5.6)	0.002
6-mo. PFS rate (95% CI)	20.8% (13.5-29.3)	13.5% (6.5-23.1)	30.2% (16.2-45.4)	
**Median OS, mo. (95% CI)**	7.1 (5.5-9.4)	6.1 (4.8-8.3)	12.6 (5.5-18.5)	0.001
1-yr OS rate (95% CI)	29.1% (19.9-38.9)	17.9% (9.3-28.8)	48.4% (29.8-64.7)	

^*^ Objective response rate and disease control rate were evaluated in patients who had measurable disease (n=91).

The P values for comparison of objective response and disease control rates were assessed using the Chi2 test.

The P value for the comparison of time to event endpoints was assessed using the log-rank test.

Abbreviations: mo., months; yr, year

### Progression-free survival

**Figure 2:**
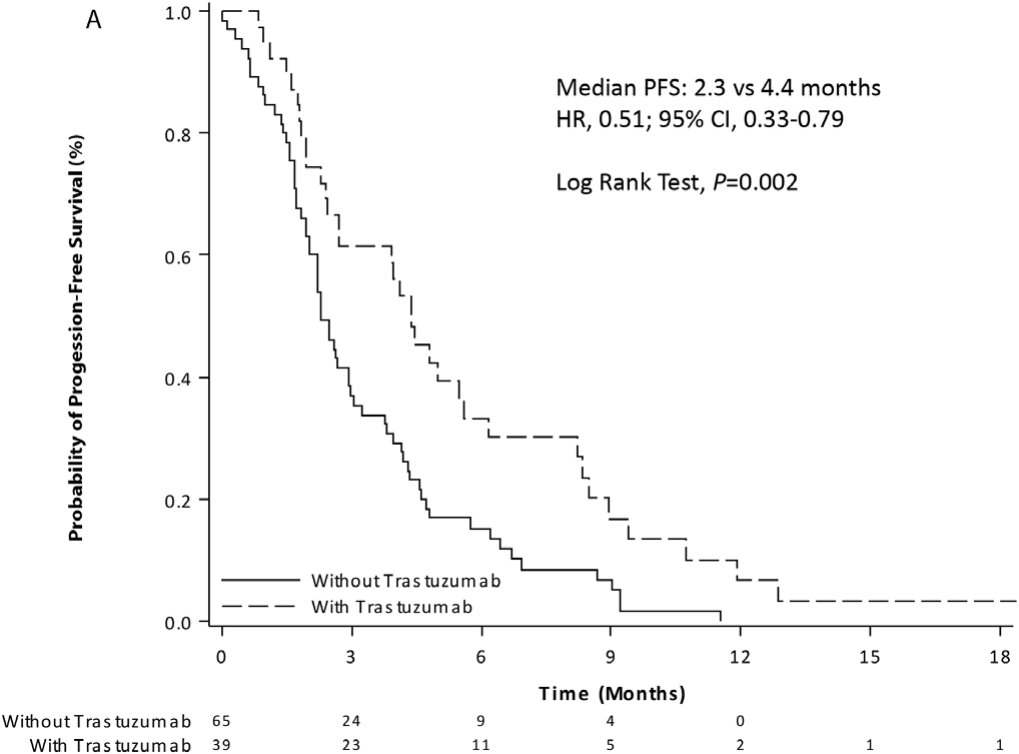

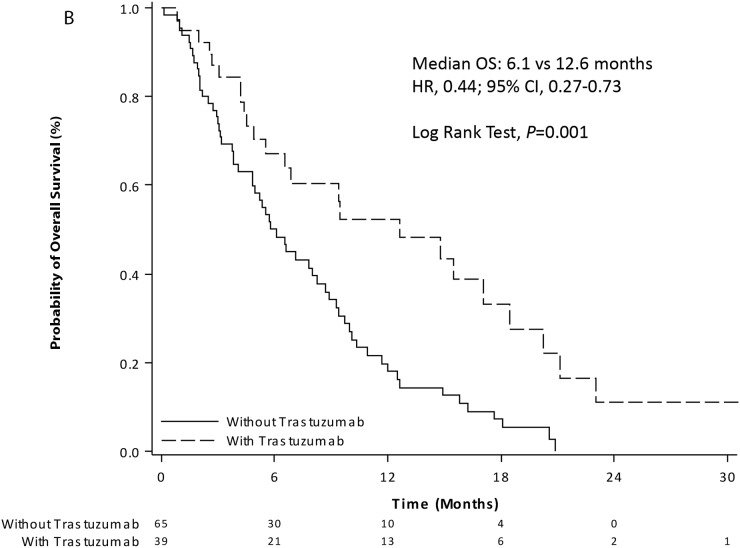
Kaplan-Meier curves of progression-free survival **(A)** and overall survival **(B)** from the start of second-line chemotherapy with (dashed line) or without (solid line) trastuzumab in patients with HER2-positive advanced gastric or gastro-esophageal junction adenocarcinoma.

In the overall population, 98 (94.2%) patients had tumor progression or died from the start of second-line chemotherapy until the end of follow-up. The median PFS was 2.7 months (95% CI, 2.3-4.0), and the 6-months PFS rate was 20.8% (95% CI, 13.5-29.3) (Table 2). The continuation of trastuzumab in second-line chemotherapy was significantly associated with an improvement of median PFS as compared to trastuzumab discontinuation (4.4 versus 2.3 months; *P*=0.002) (Figure 2A).

In univariate analysis, trastuzumab beyond progression, ECOG PS, measurable disease and the number of metastatic sites were significantly associated with PFS (Table 3). In multivariate analysis, the following factors remained significantly associated with longer PFS: trastuzumab in second-line chemotherapy (with versus without: HR, 0.56; 95% CI, 0.35-0.89; *P*=0.01), ECOG PS (0-1 versus 2: HR, 0.56; 95% CI, 0.34-0.91; *P*=0.02) and measurable disease (yes versus no: HR, 0.42; 95% CI, 0.21-0.85; *P*=0.01) (Table 3).

**Table 3:** Univariate and multivariate analysis for progression-free survival and overall survival from the second-line of chemotherapy

	Progression-Free Survival						Overall Survival					
	Univariate			Multivariate			Univariate			Multivariate		
	HR	(95% CI)	P	HR	(95% CI)	P	HR	(95% CI)	P	HR	(95% CI)	P
**Age (years)**												
≤ 60 vs > 60^R^	0.85	(0.57-1.27)	0.43				1.18	(0.76-1.82)	0.44			
**Gender**												
Male vs female ^R^	0.65	(0.40-1.05)	0.08				0.62	(0.37-1.03)	0.06			
**ECOG performance status**												
0-1 vs ≥ 2 ^R^	0.52	(0.32-0.86)	0.009	0.56	(0.34-0.91)	0.02	0.35	(0.21-0.59)	<.0001	0.34	(0.20-0.57)	<.0001
**Primary tumor site**												
Junction vs stomach ^R^	0.76	(0.50-1.13)	0.18				0.70	(0.45-1.09)	0.11			
**Histological type**												
Diffuse/mixed vs intestinal ^R^	1	(0.53-1.90)	0.99				0.82	(0.41-1.63)	0.57			
**Tumor grade**												
Well/moderate vs poor ^R^	0.69	(0.46-1.05)	0.09				0.57	(0.35-0.93)	0.06			
**Measurable disease**												
Yes vs No ^R^	0.36	(0.19-0.67)	0.001	0.42	(0.21-0.85)	0.01	0.36	(0.19-0.67)	0.001	0.39	(0.19-0.79)	0.009
**Number of metastatic sites**												
0-1 vs ≥ 2 ^R^	0.63	(0.40-1.0)	0.049	0.70	(0.43-1.13)	0.14	0.61	(0.37-0.99)	0.048	0.59	(0.34-1.02)	0.06
**Second-line chemotherapy**												
With vs without trastuzumab ^R^	0.51	(0.33-0.79)	0.002	0.56	(0.35-0.89)	0.01	0.44	(0.27-0.73)	0.002	0.47	(0.28-0.79)	0.004

Abbreviations: R, reference; HR, hazard ratio; ECOG, Eastern Cooperative Oncology Group; PFS, progression-free survival; OS, overall survival.

Multivariate analysis was performed on variables potentially predictive of the risk of disease progression or death in univariate analysis (threshold, 5%).

For PFS and OS multivariate analysis, 7 patients were excluded because they had at least one missing data among the variables selected on univariate analysis; finally, 97 patients were selected for multivariate analysis.

A *P* value < 0.05 was considered statistically significant.

### Overall survival

In the overall population, 83 (79.8%) patients had died from the start of second-line chemotherapy until the end of follow-up. The median OS was 7.1 months (95% CI, 5.5–9.4), and the 1-year OS rate was 29.1% (95% CI, 19.9-38.9) (Table 2). The continuation of trastuzumab in second-line chemotherapy was significantly associated with an improvement of median OS as compared to trastuzumab discontinuation (12.6 versus 6.1 months; *P*=0.001) (Figure 2B).

In addition to trastuzumab beyond progression, ECOG PS, measurable disease and the number of metastatic sites were significantly associated with OS in the univariate analysis (Table 3). In multivariate analysis, we observed that the following factors remained significantly associated with longer OS: trastuzumab in second-line chemotherapy (with versus without: HR, 0.47; 95% CI, 0.28-0.79; *P*=0.004), ECOG PS (0-1 versus 2: HR, 0.34; 95% CI, 0.20-0.57; *P*<0.0001) and measurable disease (yes versus no: HR, 0.39; 95% CI, 0.19-0.79; *P*=0.009) (Table 3).

## DISCUSSION

In this large multicenter study of HER2-positive gastric cancer, we observed in patients with disease progression after trastuzumab-containing platinum-based therapy that changing chemotherapy with continuation of trastuzumab was associated with improved clinical outcomes. Patients who continued trastuzumab in second-line treatment had a better ORR, and a significantly longer PFS and OS than those who stopped trastuzumab. Our data support the concept of continuing trastuzumab beyond first-line progression in HER2-positive gastric cancer, as was already demonstrated in HER2-positive breast cancer [19, 20].

There are some clinical data about second-line anti-HER2 therapies, but these studies were not dedicated to patients pretreated with trastuzumab. The TYTAN randomized phase III trial evaluated the efficacy of paclitaxel with or without lapatinib, a dual inhibitor of EGFR and HER2 tyrosine kinase activity, as second-line treatment in patients with HER2 FISH-positive tumors [21]. The addition of lapatinib to paclitaxel was not associated with an improvement of either PFS or OS [21]. In this trial, only 6% of patients received a prior treatment with trastuzumab. Lapatinib was also evaluated in first-line treatment in HER2 FISH-positive tumors in the LOGIC phase III trial comparing capecitabine-oxaliplatin (CAPOX) with or without lapatinib [22]. The addition of lapatinib to CAPOX did not significantly increase survival, even in patients with IHC3+ or IHC2+/FISH-positive tumors [22]. The GATSBY study is another randomized trial evaluating trastuzumab emtasine (T-DM1) versus taxane in second-line treatment of patients with HER2-positive tumors defined by IHC3+ or IHC2+/FISH-positive [23]. This study did not show a superiority of T-DM1 over taxane in terms of PFS and OS, regardless of whether or not the first-line treatment included trastuzumab [23]. Therefore, in contrast to HER2-positive breast cancer [20], trastuzumab remains the only anti-HER2 therapy validated in HER2-positive gastric cancer. Further elucidation of HER2 biology is needed to shed light on differences in terms of efficacy of anti-HER2 therapies between gastric and breast cancers.

Recently, Li et al evaluated the efficacy of trastuzumab beyond progression in a prospective observational cohort of 59 Chinese patients with HER2-positive advanced gastric cancer [24]. This study showed that continuing trastuzumab in combination with chemotherapy versus chemotherapy alone was associated with an improvement of PFS, while tumor response rate and OS were not significantly improved [24]. All patients received trastuzumab in combination with a first-line chemotherapy. However, in this relatively small Asian series, more than half of the patients received a non-platinum drug in first-line therapy, and approximately one-third received a platinum drug in second-line, which makes it difficult to interpret the real value of trastuzumab beyond progression after failure of standard platinum-based treatment. In 2017, Makiyama et al. presented at the ASCO GI meeting, an observational study of 85 patients from Japan treated with continuation (n=59) or not (n=25) of trastuzumab in second-line treatment. In this study, the trastuzumab beyond progression strategy was significantly associated with an increase of median OS (12.8 versus 7.9 months; HR, 0.50; 95% CI 0.29–0.84; *P*=0.01), although very poor data were given regarding the first-line treatment [25]. In addition, there is currently an ongoing randomized phase II study in Japan (WJOG7112G) comparing trastuzumab in combination with weekly paclitaxel versus weekly paclitaxel alone after failure of a trastuzumab, fluoropyrimidine and platinum containing chemotherapy (UMIN-CTR Clinical Trial ID: UMIN000009297).

In our study, the choice of second-line chemotherapy regimen was left to clinician's discretion. We observed that patients were more frequently treated with FOLFIRI (64.4%) than taxane (22.1%) in second-line chemotherapy. This finding is probably related to neuropathy induced by the first-line platinum-based chemotherapy, resulting in a limitation of taxane-based second-line treatment that also induces neuropathy. This toxicity profile may have introduced a bias in the decision to continue trastuzumab beyond progression since trastuzumab is readily combined with taxane in breast cancer, while data on combining FOLFIRI with trastuzumab are lacking. A minority of patients received a platinum-based second-line chemotherapy (n=14, 13.5%) with a different compound from that used in first-line (11 were treated with oxaliplatin after cisplatin and 3 with cisplatin after oxaliplatin). A phase II study has suggested that oxaliplatin might reverse resistance to cisplatin in gastric cancer treatment [26]. However, the synergic effect of trastuzumab beyond progression by changing the platinum salts used in first line was not evaluated in our study due to the low number of patients included in these subgroups. Of note no cardiac toxicity was reported in trastuzumab beyond progression group during the time of the study.

We observed in our study that the response rate in first-line was higher for patients treated with trastuzumab beyond progression. A better comprehension of the mechanisms leading to trastuzumab sensibility or resistance is needed to identify the patients who would benefit from continuation of trastuzumab beyond progression. Preclinical studies have suggested that activation of alternative tyrosine kinase receptors (such as EGFR, HER3, FGFR2 and MET) or signaling pathways (such as Src and Notch1), often leading to epithelial-mesenchymal transition, could lead to secondary resistance to trastuzumab [27]. Micro RNAs have also been pointed out as a potential actor in primary and secondary resistance to HER2-targetting therapy [28]. Moreover, the direct loss of HER2 overexpression or amplification have been reported after exposition to trastuzumab containing therapy [29, 30], raising the question of the interest of new biopsies at disease progression.

Despite the low rate of missing data (under 10%), the main limitation of our study is its retrospective nature and the small series of patients. Patients in the group receiving second-line chemotherapy without trastuzumab had more frequently ≥2 metastatic sites than patients who continued trastuzumab beyond progression. This might suggest that patients without trastuzumab in second-line treatment had initially more aggressive disease. Nevertheless, in the multivariate analysis including these variables (Table 3), the HR for PFS and OS was still significantly in favor of continuation of trastuzumab beyond progression. Another limitation is the small number of patients in each treatment subgroup that not allow us to evaluate which regimen of second-line chemotherapy was the most effective with trastuzumab beyond progression.

## MATERIALS AND METHODS

### Patients

This retrospective multicenter non-interventional study included, in 24 French care centers, all consecutive patients with histologically proven HER2-positive advanced (locally advanced or metastatic) gastric or GEJ adenocarcinoma who after progression on trastuzumab plus platinum-based therapy received from May 2010 to December 2015 a second-line chemotherapy with irinotecan, taxane or platinum-based therapy, with or without trastuzumab. We excluded patients who had stopped first-line chemotherapy for reasons other than disease progression. The HER2-positive tumor was defined as IHC 3+ or IHC 2+/FISH-positive status. This study was approved by the Pitié Salpêtrière Hospital ethics committee (CPP - Ile-de-France, Paris VI), and was conducted in agreement with the appropriate ethical guidelines and legislation.

### Treatment and Outcome

The choices of second-line chemotherapy regimen and the decision to continue trastuzumab or not were left to the physician's discretion. Chemotherapy was continued until disease progression or limiting toxicity. Routine clinical, laboratory and CT scan follow-up was performed every 2 or 3 months according to physician and local practice, or earlier if progression was suspected. Tumor response was assessed by CT scans in patients with measurable disease based on RECIST criteria (version 1.1) (patients without measurable disease were evaluated for PFS and OS only).

By reviewing of medical records, data were collected on relevant clinical and tumor characteristics, chemotherapy regimen received on first and second line, tumor response, date of disease progression and survival status at the last follow-up. The data were updated in June 2016.

### Statistical analyses

The main objective of this study was to evaluate the impact on PFS of continuing or not trastuzumab in second-line chemotherapy. Baseline clinical and pathological characteristics were compared according to the continuation or not of trastuzumab in second-line chemotherapy. Continuous variable were described as means, standard deviation, median and range, and compared using Student test or Wilcoxon test in case of normal distribution or not, respectively. Qualitative variables were described as frequencies and percentages and compared using the χ^2^ test or Fisher's exact test as appropriate. The median follow-up was estimated using the reverse Kaplan-Meier method.

Progression-free survival was defined as the time elapsed from the first cycle of second-line chemotherapy until the date of first progression or death (all causes), whichever came first. Alive patients without disease progression were censored at the last follow-up date. Overall survival was defined as the time elapsed from the first cycle of second-line chemotherapy until death (all causes). Alive patients were censored on the last follow-up date. Survival curves were estimated using the Kaplan-Meier method and compared using the log-rank test. Cox univariate and multivariate analysis were used to calculate HR with a 95% CI. The variables with *P* values ≤0.05 in univariate analysis were eligible for the Cox multivariable regression model. The assumption of risks proportionality and log-linearity were verified for each variable. Correlations between all variables were explored. In case of strong correlation between two variables, either one variable was included in the multivariate model.

A *P* value of less than 0.05 was considered statistically significant. All statistical tests were two-sided. All analyses were performed using SAS software version 9.4 (SAS Institute, Cary, NC, USA).

## CONCLUSIONS

Our study suggested a significant improvement of PFS and OS with continuation of trastuzumab in second-line chemotherapy for patients with HER2-positive advanced gastric or GEJ adenocarcinoma. The results support the concept of continuing the inhibition of the HER2 signaling pathway beyond progression, as already demonstrated in breast cancer. The low rate of HER2-positive status in gastric cancer should encourage the development of international collaborations in order to confirm the utility of trastuzumab beyond progression through a large randomized phase III study.
